# Improved Sixty-Day Mortality in Candidemia with Antifungal Treatment Within 72 Hours of Fever Onset: A Single-Center Retrospective Study in Rural Japan

**DOI:** 10.3390/idr17020036

**Published:** 2025-04-21

**Authors:** Koji Hayashi, Chizuru Hashimoto, Kohei Ueda, Yuka Nakaya, Asuka Suzuki, Maho Hayashi, Mamiko Sato, Yasutaka Kobayashi

**Affiliations:** 1 Department of Rehabilitation Medicine, Fukui General Hospital, 55-16-1 Egami, Fukui City 910-8561, Fukui, Japansatomoko@f-gh.jp (M.S.); 2 Department of Infection Control Team, Fukui General Hospital, 55-16-1 Egami, Fukui City 910-8561, Fukui, Japan; 3 Department of Internal Medicine, Fukui General Hospital, 55-16-1 Egami, Fukui City 910-8561, Fukui, Japan; 4 Graduate School of Health Science, Fukui Health Science University, 55-13-1 Egami, Fukui City 910-3190, Fukui, Japan; yasutaka_k@fukui-hsu.ac.jp

**Keywords:** Candida, candidemia, prognosis, antifungal drug, echinocandins, central venous catheter

## Abstract

**Introduction:** Prognostic factor investigations for candidemia have been conducted in large-scale facilities, leading to significant evidence, including early administration of echinocandin antifungal agents and removal of central venous catheters (CVCs). In departments that provide aggressive chemotherapy or transplantation, candidiasis markers are regularly evaluated, and preemptive treatments may be initiated. However, in resource-limited facilities, candidemia detection largely relies on vital signs like fever and blood cultures. This study assessed whether evidence from large-scale facilities applies to such settings. Additionally, while prior studies indicate that early antifungal treatment is based on positive blood cultures, no established criteria exist for early administration based on fever as an indicator. **Methods:** This study analyzed cases of candidemia from blood cultures at Fukui General Hospital (2014–2024). Patients aged 18 or older with at least one positive blood culture for *Candida* species and clinical signs of infection were included, while contamination cases were excluded. The patients were categorized into survival and death groups based on 60-day survival from fever onset. The variables collected included age, gender, duration from admission to fever onset, time from fever onset to blood culture collection and antifungal treatment initiation, antifungal treatment within 72 h, serum albumin levels, history of cancer, diabetes, empiric echinocandin treatment, CVC insertion, duration of CVC insertion until fever onset, use of total parenteral nutrition, broad-spectrum antibiotic use, and sequential organ failure assessment (SOFA) score. Fever was defined as a body temperature of 38.0 °C or higher, guiding blood culture collection. **Results:** Of 30 candidemia cases, 29 were analyzed. Survival was significantly associated with younger age (average 73.3 ± 13.3 vs. 83.1 ± 9.1 years, *p* = 0.038) and antifungal treatment within 72 h of fever onset (9 vs. 3, *p* = 0.025). CVC use was of marginal significance (8 vs. 13, *p* = 0.108). There was a significant difference in the duration (in days) of CVC insertion until fever onset (median [IQR]: 15.5 [11.75–19.5] vs. 30.0 [19.0–39.0], *p* = 0.027). Logistic regression identified early antifungal treatment (OR = 0.065, *p* = 0.035) and CVC use (OR = 21.8, *p* = 0.024) as independent predictors of mortality. **Conclusions:** Early antifungal treatment within 72 h of fever onset and CVC use were independent predictors of mortality in candidemia. The importance of early antifungal treatment was reaffirmed even in smaller facilities. The impact of CVC insertion on 60-day survival cannot be readily generalized due to the limited sample size. Further research is needed to clarify the impact of fever-based antifungal initiation and CVC use on 60-day survival.

## 1. Introduction

Invasive fungal infections have been a major threat to humans over the past three decades [1]. The incidence of invasive fungal infections in the United States has been reported as 27.2 cases per 100,000 patients per year, with *Candida* infections accounting for 55% of the cases [2]. Although reports vary, mortality from candidemia remains high, ranging from 35% to 67% [3,4,5].

The Infectious Diseases Society of America (IDSA) published the Clinical Practice Guideline for the Management of Candidiasis in 2016, compiling evidence for the treatment of *Candida* infections. This guideline emphasizes several management strategies for candidemia, including the administration of echinocandins as the initial therapy and considering early removal of the central venous catheter (CVC) if the cause is suspected to be the CVC and it can be safely removed [6]. In addition, early treatment with echinocandins administered within 72 h of a positive *Candida* blood culture has also been shown to be effective [7].

While previous studies have analyzed the prognosis of candidemia based on time to positive blood culture, these analyses may be limited by the potential delay between the onset of candidemia and blood culture collection. This study aimed to assess the benefits of early antifungal drug administration for candidemia by using the onset of fever (≥38 °C) as a more precise marker for the initiation of infection.

To the best of our knowledge, there are no reports in the English literature that have examined the incidence and prognostic factors of candidemia in rural hospitals without sterile rooms or active anticancer chemotherapy departments, such as medical oncology, hematology, pulmonary medicine, or transplant surgery. In the hematology department, particularly within units administering active anticancer chemotherapy, fungal infections are monitored periodically, including evaluation for β-d-glucan and antigen tests. In such facilities, the early detection of candidemia or other fungal infections may lead to pre-emptive treatment with antifungal drugs. In facilities without such units, candidemia is typically detected through vital sign monitoring, blood tests, and blood cultures. Therefore, the detection of candidemia tends to be delayed in these latter facilities. This study aimed to conduct a prognostic analysis of candidemia in less-equipped facilities, providing valuable evidence to complement existing research from large-scale facilities.

## 2. Materials and Methods

### 2.1. Study Design and Data Collection

This study included cases of fungi isolated from blood cultures at Fukui General Hospital, a 315-bed secondary care center, between 1 January 2014 and 31 December 2024. Blood cultures were processed using an automated analyzer with both aerobic and anaerobic culture bottles. Patients aged 18 or older with candidemia, defined as at least one positive blood culture for *Candida* species along with clinical signs and symptoms of infection, were included. Cases of contamination were excluded. Cases of contamination were defined as those in which only one out of two sets of blood cultures tested positive, and the attending physician judged it to be contamination. The patients were categorized into two groups based on 60-day survival from fever onset: the survival group and the death group.

The following variables were collected from medical records: age, gender, duration from admission to fever onset (in days), duration from fever onset to collect blood cultures (in hours), duration from fever onset to initiation of antifungal treatment (in hours), antifungal treatment initiated within 72 h of fever onset (Y/N), serum albumin levels, history of cancer (Y/N), diabetes (Y/N), empiric echinocandin antifungal treatment (Y/N), insertion of a CVC (Y/N), duration of CVC use until fever onset (in days), use of total parenteral nutrition (Y/N), history of broad-spectrum antibiotic use (Y/N), and sequential organ failure assessment (SOFA) score. Although fever can be defined in various ways, for this study, it was defined as a body temperature of 38.0 °C or higher, which served as a guide for blood culture collection in our hospital.

### 2.2. Statistical Analysis

Statistical analysis was performed on the collected variables. As this was a small-scale study, a significance level of *p* < 0.05 was used. However, given the limited sample size, *p*-values below 0.15 were considered to indicate a trend towards significance, as conventional thresholds might overlook meaningful associations that could have clinical or practical relevance. Categorical variables were analyzed using Fisher’s exact test. Continuous variables were assessed for normality using the Shapiro–Wilk test and for homogeneity of variances using the F-test. A two-sample *t*-test was employed for normally distributed groups with equal variances, while the Mann–Whitney U test was used for other comparisons. Logistic regression analysis was conducted to examine the association between 60-day survival (survival or death) and variables that demonstrated statistical significance and marginal significance (*p* < 0.15), along with age, which are well-known risk factors for candidemia mortality. If there was a significant difference or marginal significance (*p* < 0.15) in 60-day survival from candidemia with respect to both the categorical variable of whether antifungal drugs were administered within 72 h and the continuous variable of the time of antifungal drug administration, the administration of antifungal drugs within 72 h was included as a covariate. Similarly, if a significant difference or marginal significance (*p* < 0.15) was found in 60-day survival from candidemia regarding the categorical variable of CVC use and the continuous variable of CVC duration until fever onset, the use of CVC was included as a covariate. All analyses were performed using R software (version 4.4.1, R Foundation for Statistical Computing, Vienna, Austria).

## 3. Result

**Figure 1:**
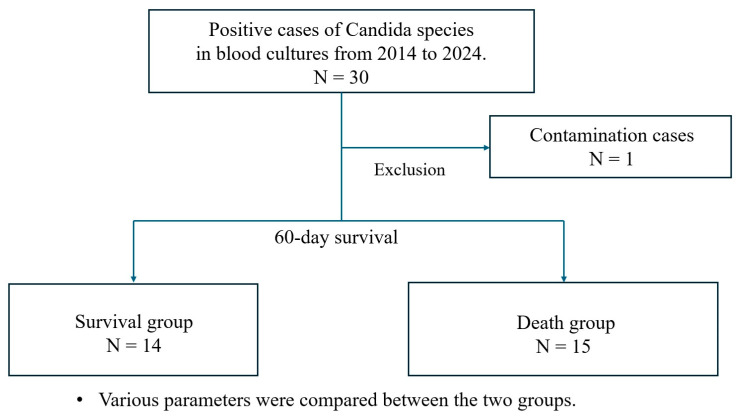
Study design. This study included patients with Candidemia (defined as at least one positive blood culture for *Candida* species along with clinical signs and symptoms of infection) who were treated at Fukui General Hospital, a 315-bed secondary care center, between 1 January 2014 and 31 December 2024. Blood cultures were processed using an automated analyzer with both aerobic and anaerobic culture bottles. Cases of contamination were excluded (defined as only one positive blood culture out of two sets, with attending physician confirmation of contamination). The patients were categorized into two groups based on 60-day survival from fever onset: the survival group and the death group.

A total of 30 cases among a total of 57,919 hospitalized patients had positive blood cultures during the study period. One case was excluded due to contamination. Of the remaining 29 cases, 14 were classified into the survival group and 15 into the death group (Figure 1). Table 1 shows the characteristics of both groups. The *Candida* species included eight *C. albicans*, five *Nakaseomyces glabratus* (*N. glabratus*, formerly known as *C. glabrata)*, five *C. parapsilosis*, six *C. tropicalis*, and five other *Candida* species (unidentified or multiple species). All isolated *Candida* species were micafungin-susceptible (MIC ≤ 1), while azole resistance was limited to *N. glabratus*, with all *N. glabratus* isolates exhibiting MIC ≥ 4. Two cases received no antifungal treatment in the death group, while the remaining 27 cases were treated. All cases of echinocandin use, including empirical and definitive therapy, employed micafungin. In four cases, the antifungal agent initially selected for empirical therapy was changed to another antifungal agent. Two fatal cases (*C. tropicalis* and *N. glabratus*) switched from fluconazole to micafungin, while two surviving cases (*C. albicans* and *C. parapsilosis*) switched from micafungin to fluconazole. Broad-spectrum antibiotic treatment was used in 26 cases.

When divided into two groups based on prognosis, age was assessed to follow a normal distribution. The Shapiro–Wilk normality test yielded *p* = 0.52 for the survival group and *p* = 0.099 for the non-survival group. Additionally, the F-test for homogeneity of variances yielded *p* = 0.17, indicating equal variances could be assumed. Regarding duration from admission to fever onset (in days), the Shapiro–Wilk normality test confirmed a normal distribution, with *p* = 0.10 for the survival group and *p* = 0.52 for the non-survival group. Similarly, the F-test indicated equal variances with *p* = 0.91. Regarding serum albumin levels, the Shapiro-Wilk normality test confirmed a normal distribution, with *p* = 0.97 for the survival group and *p* = 0.90 for the non-survival group. Similarly, the F-test indicated equal variances with *p* = 0.90. Therefore, a *t*-test was applied for the analysis of age, duration from admission to fever onset, and serum albumin levels. In contrast, for the duration from fever onset to blood culture collection (in hours), the Shapiro–Wilk normality test yielded *p* = 0.000066 for the survival group and *p* = 0.000025 for the non-survival group, indicating that normality could not be assumed. Consequently, the Mann–Whitney U test was applied. Similarly, for the duration from fever onset to the initiation of antifungal treatment (in hours), the Shapiro–Wilk test results were *p* = 0.0000085 for the survival group and *p* = 0.89 for the non-survival group. Additionally, for the duration of CVC until fever onset (in days), the Shapiro–Wilk test results were *p* = 0.70 for the survival group and *p* = 0.00026 for the death group. Based on these results, the Mann–Whitney U test was also applied to these variables.

The results of the statistical analysis between the two groups are shown in Table 1. As a result of the *t*-test, the survival group was significantly younger (average 73.3 ± 13.3 vs. 83.1 ± 9.1 years, *p* = 0.038), whereas serum albumin did not show a statistically significant correlation (average 2.17 ± 0.57 vs. 2.04 ± 0.54). The results of the Mann–Whitney U test indicated no significant correlation in the duration (hours) from fever onset to blood cultures between the two groups (median [IQR]: 2.29 [0.84–30.34] vs. 2.22 [0.04–30.92], *p* = 0.84). However, there was marginal significance in the duration (hours) from fever onset to antifungal treatment (median [IQR]: 55.7 [47.5–95.3] vs. 89.0 [75.5–147.7], *p* = 0.065). Additionally, there was a significant difference in the duration (in days) of CVC insertion until fever onset (median [IQR]: 15.5 [11.75–19.5] vs. 30.0 [19.0–39.0], *p* = 0.027).

Fisher’s exact test revealed a significant association between 60-day mortality in candidemia and antifungal treatment within 72 h of fever onset (*p* = 0.025). In addition, the use of CVC was evaluated and found to have marginal significance (*p* = 0.108). No significant associations were found between the two groups for the following factors: duration from admission to fever onset (*p* = 0.41), current or past cancer (*p* = 0.71), diabetes (*p* = 1.0), empiric echinocandin antifungal treatment (*p* = 1.0), use of total parenteral nutrition (*p* = 1.0), history of broad-spectrum antibiotic use (*p* = 1.0), SOFA score (*p* = 0.19), and detection of *C. albicans* (*p* = 0.43).

Table 2 presents the results of the logistic regression analysis incorporating antifungal treatment administered within 72 h of fever onset, which showed a statistically significant difference. The analysis indicated that antifungal treatment within 72 h from fever onset (OR = 0.065, 95% CI = [0.0027–0.62], *p* = 0.035) and CVC use (OR = 21.8, 95% CI = [2.09, 604.8], *p* = 0.024) were independently significant prognostic factors.

## 4. Discussion

This study was conducted to address two limitations of the existing prognostic analyses of candidemia. First, most previous studies were conducted in facilities with sterile units or those actively performing anticancer chemotherapy, making their findings not readily generalizable to hospitals without such facilities or departments. In well-equipped facilities, such as those with sterile rooms and microbial identification tools, the identification of fungi is significantly faster and more accurate compared to less-equipped facilities. For example, in clinical settings with sterile rooms, there is a system in place to routinely evaluate fungal markers and immediately administer antifungal medications if any abnormalities are detected. Additionally, if equipment like mass spectrometry is available, it can lead to the identification of infectious microorganisms on the same day. Therefore, evidence generated in highly equipped facilities might not be readily applicable to those with fewer resources. Second, although blood cultures are likely to be drawn promptly in most large centers, previous studies often focused on early antifungal administration based on the time to positive blood culture, which may not accurately reflect the onset of candidemia, depending on the timing of blood culture collection.

This study was conducted at a single center in rural Japan. Due to the lack of mass spectrometry and genetic testing for fungal identification at our facility, traditional blood cultures were the sole method used for detection. Additionally, our center primarily comprises departments, such as general internal medicine, general surgery, orthopedics, and rehabilitation, and lacks specialized units like hematology/oncology or pulmonary medicine that frequently administer chemotherapy, which addresses the first limitation. Furthermore, this study aimed to address the second limitation by analyzing the prognosis of candidemia in relation to early antifungal drug administration based on fever onset. Since no studies have examined the prognosis of candidemia in relation to fever onset, we aimed to verify a 60-day survival rate when antifungal drugs are administered within 72 h of fever onset. The statistical analysis revealed that the administration of antifungal drugs within 72 h of fever onset was an independent factor associated with a favorable prognosis. While the limited sample size and wide 95% confidence intervals warrant caution in interpreting the odds ratios, the findings are supported by the well-established favorable factors associated with early antifungal therapy for candidemia [7,8].

Reported risk factors for developing candidemia include abdominal surgery, steroid use, parenteral nutrition, advanced age, long-term use of broad-spectrum antibiotics, immunosuppressant use, cancer chemotherapy, neutropenia, trauma, CVC, multiple *Candida* colonizations, renal replacement therapy, prolonged ICU stays, mechanical ventilation, diabetes, high APACHE-II scores, malnutrition, and burns [9]. Additionally, candidemia is associated with increased mortality due to challenges such as difficulty in catheter removal, inappropriate antifungal drug use, ventilator dependence, severe underlying diseases (higher Charlson scores), advanced age, multiple organ failure, severe sepsis or septic shock, and concurrent bacterial infections [3,10,11,12].

Furthermore, many studies support the effectiveness of echinocandins in treating candidemia [7,8,13]. The empirical use of echinocandins, which are a class of antifungal drugs that inhibit the synthesis of β-glucan in the fungal cell wall via noncompetitive inhibition of the enzyme 1,3-β glucan synthase [14,15,16], has been reported to significantly reduce overall mortality in candidemia [13]. Early treatment with echinocandins has also been shown to be effective [7,8]. Specifically, administering caspofungin within 72 h of a positive *Candida* blood culture significantly improves outcomes compared to later administration [7]. Moreover, administering anidulafungin within 6 h to patients with sepsis, with or without septic shock and bacterial pneumonia in the ICU, has been shown to significantly reduce the 30-day all-cause mortality rate [8].

CVC management significantly influences the prognosis of candidemia. Patients with retained CVCs have a higher risk of death compared to those who undergo CVC removal [17,18]. While the IDSA guidelines recommend removing CVCs as early as possible [6], some studies have shown that CVC removal at any time is strongly linked to improved outcomes, while early removal (within 24 or 48 h) does not necessarily yield clinical benefits [19,20]. Although an EQUAL *Candida* score of less than 10 is considered to predict poor prognosis, data suggest that patients without CVCs have lower EQUAL *Candida* scores than those with CVCs [20,21]. This may be explained by the fact that patients without CVCs generally have less severe disease and a lower mortality rate [22].

In our center, all the patients were malnourished (with hypoalbuminemia) and almost all had received broad-spectrum antibiotics. Additionally, many of the patients were elderly and had a CVC inserted. Hence, our study’s findings regarding the risks associated with the development of candidemia were consistent with those of previous studies. In our study, while age was found to significantly increase mortality in the univariate analysis, it was not identified as an independent risk factor in the logistic regression analysis. However, advanced age is a well-known risk factor for the development of candidemia and associated mortality [3,9,10,11,12]. In addition, we estimated that the increase in mortality in older patients with candidemia may be related to the tendency of older patients to have more serious or a higher number of underlying diseases. Therefore, the small number of cases may have contributed to the lack of an independent prognostic factor. Additionally, we evaluated the SOFA score as a surrogate marker of disease severity in our study. The SOFA score is a widely used marker of sepsis and organ dysfunction that assists in the evaluation of the clinical condition of the individual patient [23]. The median SOFA score was 5.0 in the death group compared to 3.5 in the survival group, indicating that the SOFA score was higher in the death group. However, there was no significant correlation between the two groups. This result may also be attributed to the small sample size.

A unique finding in our study was that the administration of antifungal drugs (regardless of type) within 72 h of the initial fever was significantly associated with reduced mortality. In contrast to previous reports, which used the timing of positive blood cultures as a criterion, our approach avoids being heavily influenced by factors such as the timing of blood collection and serum fungal load, which can vary independently of the patient’s condition. Fever is a clinically significant and easily interpretable indicator. Using fever as a criterion highlights the importance of performing blood cultures and initiating empirical antifungal treatment early. However, as previous studies suggest, our study populations might have had a better prognosis if an echinocandin, rather than an azole, had been used early in empirical treatment. One reason for this is the increasing isolation of azole-resistant *Candida* strains, including *N. glabratus* [24]. Indeed, *N. glabratus* cases were frequently isolated from the poor prognosis group in our study.

Regarding the poor prognosis in the CVC insertion group, previous studies have not demonstrated an association with increased mortality, although CVC insertion is linked to a heightened risk of developing candidemia. *Candida* is known to form biofilms on CVCs and can become resistant to antifungal drugs or lead to prolonged infections, which is one of the reasons why CVC removal is recommended [25]. Furthermore, our study revealed that long-term CVC use was a significant risk factor for 60-day mortality in candidemia. Therefore, the insertion of a CVC, particularly for long-term use, may be a risk factor associated with 60-day mortality. On the other hand, in our study, the insufficient number of cases made it difficult to assess the risk related to catheter removal. Additionally, although the CVC insertion group was statistically evaluated as an independent prognostic factor, caution is warranted when interpreting this result due to the wide 95% confidence interval.

A strength of this study is that using fever as an indicator makes this study’s approach straightforward and easy to understand. Additionally, this study emphasizes the importance of promptly collecting blood cultures immediately after the onset of fever. Delays in this process can lead to delays in antifungal drug administration, potentially resulting in a poor prognosis. Moreover, this study, like previous reports, identified a high proportion of azole-resistant *Candida* species, including *N. glabratus*, underscoring the need for the early use of echinocandin antifungals.

However, there are some limitations in this study. Firstly, the small sample size restricts the generalizability of our findings. While we identified CVC insertion as a significant factor affecting 60-day survival, the limited number of cases necessitates caution in drawing definitive conclusions. Further research with larger populations is needed to thoroughly examine the impact of early antifungal treatment initiated within three days of fever onset, as well as the role of CVC insertion in 60-day mortality. Secondly, our study design cannot detect candidemia in patients who are afebrile or have only a slight fever below 38 °C. It is well known that fever may be absent in severe sepsis cases [26]. Future research should analyze prognostic factors of candidemia, including afebrile candidemia, using a prospective study design.

## 5. Conclusions

Our study reaffirms the previously established evidence regarding the importance of early antifungal drug administration as a significant prognostic factor for mortality from candidemia. Importantly, our findings suggest that this evidence is applicable to small-sized facilities that do not have sterile rooms or actively conduct chemotherapy, highlighting the need for prompt intervention in these settings as well.

While our results indicate an association between CVC insertion and mortality, the small sample size limits our ability to draw definitive conclusions regarding this factor. Consequently, caution should be exercised when interpreting these results, and further research with larger populations is required to clarify the impact of fever-based antifungal initiation and CVC insertion on outcomes in patients with candidemia.

## Figures and Tables

**Table 1:** The characteristics of the survival and death groups.

Parameters	Survival Group in 60 Days	Death Group in 60 Days	*p*-Value
	N = 14	N = 15	
Age, years (Average)	73.3 ± 13.3	83.1 ± 9.1	0.038 **
Gender, male	8	10	0.5
Duration (days) from admission to fever onset (Median (IQR))	33.0 (11.3–55.1)	39.0 (27.9–64.7)	0.41
Duration (hrs) from fever onset to blood cultures (Median (IQR))	2.29 (0.84–30.34)	2.22 (0.04–30.92)	0.84
Blood cultures within 12 hrs from fever onset (Yes)	10	8	0.34
Duration (hrs) from fever onset to antifungal treatment (Median (IQR))	55.7 (47.5–95.3)	89.0 (75.5–147.7)	0.065 *
Antifungal treatment within 72 hrs from fever onset (Yes)	9	3	0.025 **
Serum albumin level (Average)	2.17 ± 0.57	2.04 ± 0.54	0.55
Current cancer or past cancer (Yes)	5	7	0.71
Diabetes (Yes)	5	6	1.0
Empiric echinocandin antifungals treatment (Yes)	9	9	1.0
Use of central venous catheter (Yes)	8	13	0.108 *
Duration (days) of central venous catheter use until fever onset (Median (IQR))	15.5 (11.75–19.5)	30.0 (19.0–39.0)	0.027 **
Use of total parenteral nutrition (Yes)	8	8	1.0
History of using broad-spectrum antibiotics (Yes)	13	13	1.0
Sequential organ failure assessment (SOFA) score (Median (IQR))	3.5 (2.3–4.0)	5.0 (3.5–7.0)	0.19
*C. albicans* (Yes)	5	3	0.43
Types of fungi			
*C. albicans*	5	3	-
*Nakaseomyces glabratus (C. glabrata)*	0	5	-
*C. parapsilosis*	4	1	-
*C. tropicalis*	3	3	-
Others (unidentified or positive for multiple fungi)	2	3	-

* Statistical marginal significance (0.05 < *p* ≤ 0.15). ** Statistical significance (*p* < 0.05).

**Table 2:** The results of the logistic regression analysis.

Parameter	Odds Ratio	95%CI	*p*-Value
Age	1.06	[0.97, 1.18]	0.25
Antifungal treatment within 72 hrs from fever onset (Yes)	0.065	[0.0027, 0.62]	0.035 **
Use of central venous catheter (Yes)	21.8	[2.09, 604.8]	0.024 **

** Statistical significance (*p* < 0.05).

## Data Availability

The datasets generated during and/or analyzed during the current study are not publicly available due to privacy concerns. However, they are available from the corresponding author, Koji Hayashi, upon reasonable request at kjhayashi@f-gh.jp.
